# Geraniol attenuates virulence factors by inhibiting quorum sensing of *Pseudomonas aeruginosa*

**DOI:** 10.3389/fmicb.2023.1190619

**Published:** 2023-04-27

**Authors:** Wen-Ru Li, Tao-Hua Zeng, Zhi-Qing Zhang, Qing-Shan Shi, Xiao-Bao Xie

**Affiliations:** Key Laboratory of Agricultural Microbiomics and Precision Application (MARA), Guangdong Provincial Key Laboratory of Microbial Culture Collection and Application, Key Laboratory of Agricultural Microbiome (MARA), State Key Laboratory of Applied Microbiology Southern China, Institute of Microbiology, Guangdong Academy of Sciences, Guangzhou, China

**Keywords:** quorum sensing, geraniol, *Pseudomonas aeruginosa*, virulence genes, biofilm

## Abstract

*Pseudomonas aeruginosa* is a ubiquitous opportunistic pathogen that can cause severe respiratory tract infections. Geraniol, a chemical component of essential oils, has antimicrobial and anti-inflammatory activities, along with low toxicity. However, the effect and mechanism of geraniol against *P. aeruginosa* virulence factors are rarely studied. In this study, we investigated the quorum sensing (QS) inhibitory effects and mechanisms of geraniol against *P. aeruginosa* PAO1, using physiological and biochemical techniques, quantitative reverse transcription polymerase chain reaction, and transcriptomics. Geraniol slightly affected *P. aeruginosa* PAO1 growth, prolonged the lag phase, and delayed growth periods in a concentration-dependent manner. Geraniol inhibited three QS systems of *P. aeruginosa*, *las*, *rhl*, and *pqs* by suppressing the expression level of their key genes, including the three signal synthetase encoding genes of *lasI*, *rhlI*, and *pqsABCDEH*, and the corresponding signal receptor encoding genes of *lasR*, *rhlR*, and *pqsR*. Geraniol also suppressed certain virulence genes regulated by these three QS systems, including *rhlABC*, *lasAB*, *lecAB*, *phzABMS*, and *pelABG*, resulting in the attenuation of the related virulence factors, rhamnolipids, exoprotease LasA, elastase, lectin, pyocyanin, and biofilm. In conclusion, geraniol can suppress the virulence factors of *P. aeruginosa* PAO1 by inhibiting the three QS systems of *las*, *rhl*, and *pqs*. This study is significant for improving the treatment of bacterial infections caused by *P. aeruginosa*.

## Introduction

**Figure 1:**
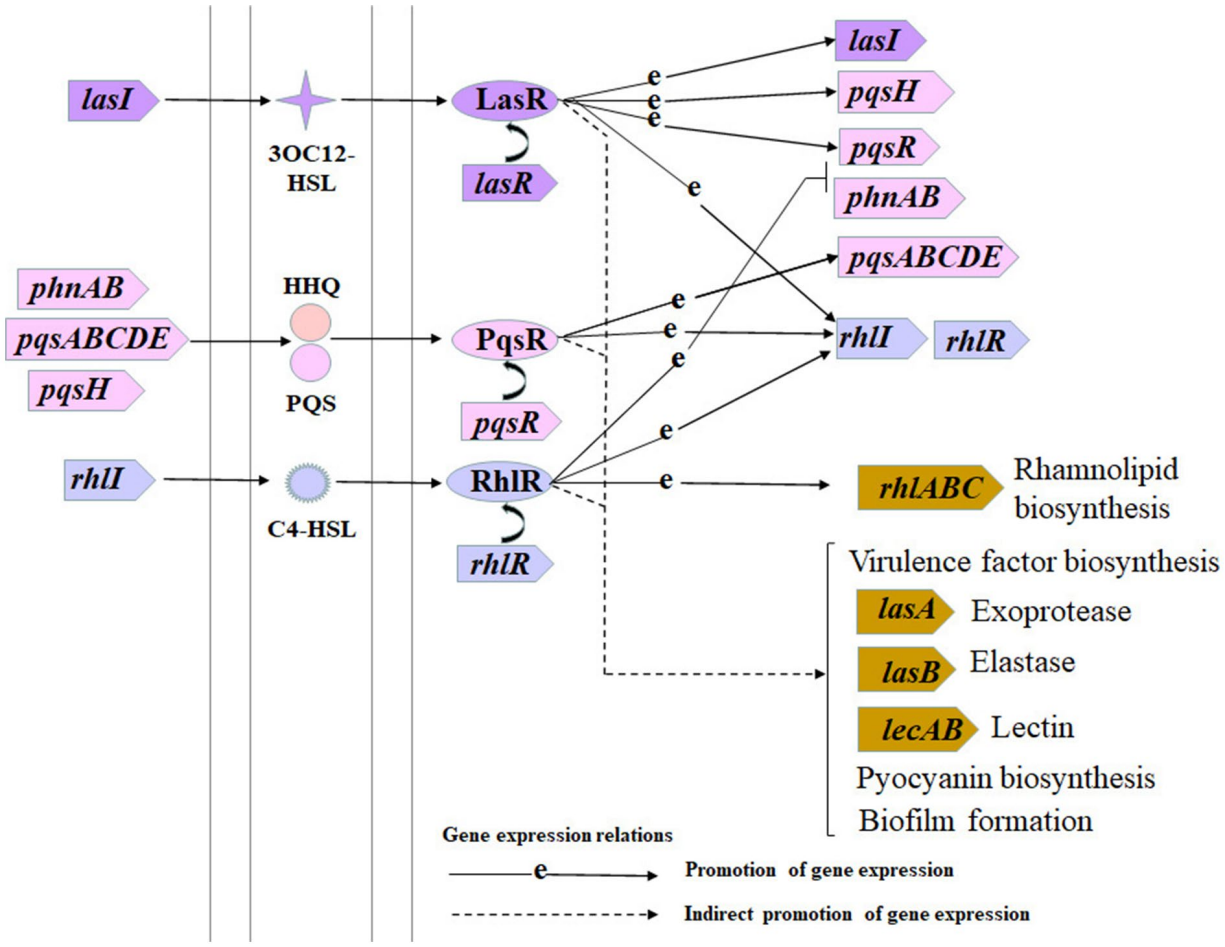
Schematic diagram of *Pseudomonas aeruginosa* hierarchical quorum sensing systems. This schematic diagram is drawn based on the quorum sensing pathway diagram from the Kyoto Encyclopedia Genes and Genomes database.

*Pseudomonas aeruginosa* is a common opportunistic pathogen that can cause severe respiratory tract infections, especially in convalescent patients with low immunity (Deshpande and Zou, 2020). *P. aeruginosa* can easily develop antibiotic resistance, which poses a challenge to clinical treatment, mainly because it has a large 6.3 Mb genome and can make adaptive responses to environmental stresses (Jurado-Martín et al., 2021; Li et al., 2022). The pan-drug resistance of *P. aeruginosa* makes it difficult to eradicate infections using traditional antibiotics. Studies have shown that bacterial quorum sensing (QS) inhibitors can solve this problem (Chatterjee et al., 2016). QS is a density dependent bacterial cell–cell communication mechanism, which is carried out by small endogenous synthetic organic molecules called autoinducers, which were discovered by Nealson et al. in 1970 (Nealson et al., 1970; Shah et al., 2021; Striednig and Hilbi, 2022). *P. aeruginosa* has three QS systems as shown in Figure 1, *las*, *rhl*, and *pqs*, which jointly regulate the production of various virulence factors (Lee and Zhang, 2015; Ahator and Zhang, 2019). The *las* and *rhl* systems are mediated by acyl-homoserine lactone signals (Li Y. et al., 2021). In the *las* system, *lasI* encodes the LasI signal synthase to produce 3-oxo-dodecanoyl-homoserine lactone (3OC_12_-HSL), and *lasR* encodes the LasR signal receptor protein. Similarly, in the *rhl* system, *rhlI* encodes the signal synthase RhlI to produce butanoyl-homoserine lactone (C_4_-HSL), and *rhlR* encodes the RhlR signal receptor protein (Li Y. et al., 2021). In the *pqs* system, genes of *pqsABCDEH* encode 2-heptyl-3-hydroxy-4(1H)-quinolone (*Pseudomonas* quinolone signal, PQS), and 2-heptyl-4-hydroxyquinoline (HHQ) signals, and *pqsR* encodes the PqsR signal receptor protein (Murray et al., 2022). The *las* system is typically located at the top of the QS hierarchy of *P. aeruginosa*, positively regulating the *pqs* and *rhl* systems; the *pqs* system connects the *las* and *rhl* systems, positively regulates the *rhl* system, and is regulated by the *rhl* system, which is located at the bottom of the QS hierarchy and is positively regulated by the *las* and *pqs* systems (Lee and Zhang, 2015; Cornelis, 2020; Soto-Aceves et al., 2021).

Furthermore, *P. aeruginosa* can cause severe infections in immunocompromised patients owing to its ability to secrete numerous extracellular virulence factors (Coin et al., 1997). As shown in Figure 1, some virulence factor biosynthesis is indirectly regulated by *P. aeruginosa* QS systems. Elastase LasB and exoprotease LasA are two virulence proteins in the *P. aeruginosa* secretome. Elastase can hydrolyze many host proteins, especially elastin, causing host tissue injury and immune response failure (Galdino et al., 2019; Everett and Davies, 2021). Although LasB can degrade elastin on its own, LasA contributes to elastin degradation (Varjani and Upasani, 2019). *lasB* is directly-positively regulated by both the *las* and *rhl* systems (Schuster and Greenberg, 2007), and indirectly regulated by the *pqs* system (Calfee et al., 2001). Pyocyanin is a characteristic *P. aeruginosa* virulence factor, causing many types of cellular damage, such as cell respiration suppression, calcium homeostasis disruption, and catalase inactivation (Li W. R. et al., 2021; Manisha et al., 2022). Bacterial lectins, such as LecA, are multivalent sugar-binding proteins that target the human glycome to act as invasion factors that damage host epithelial cells (Bodenberger et al., 2018; Day et al., 2019; Patil et al., 2022). Rhamnolipids are glycolipids mainly produced by *Pseudomonas* species and have multiple roles in metabolite uptake, host invasion, and microbial competition (Varjani and Upasani, 2019; Gdaniec et al., 2022). *P. aeruginosa* biofilms regulated by QS systems have greater antibiotic tolerance than their planktonic counterparts and usually cause chronic clinical infections that are difficult to treat (Cendra and Torrents, 2021). Therefore, *P. aeruginosa* QS systems are potential new targets to control its virulence and pathogenicity.

Geraniol, a cyclic monoterpene alcohol, is a chemical component of a large number of essential oils such as citronella, lime, lemongrass, and lavender (Kannappan et al., 2019). Geraniol has various biological activities, including anti-inflammatory, antimicrobial, antitumor, antioxidant, hepatoprotective, cardioprotective, and neuroprotective effects, and thus has been widely used in pharmaceuticals, cosmetics, and household products due to its low toxicity and environmentally friendly characteristics (Chen and Viljoen, 2022). Geraniol has been shown to exhibit high QS inhibitory activity against *P. aeruginosa* in our research. In this study, we investigated the QS inhibitory effects and mechanisms of geraniol against *P. aeruginosa* PAO1, using physiological and biochemical techniques, quantitative reverse transcription polymerase chain reaction (qRT-PCR), and transcriptomics. This study is significant for improving the treatment of *P. aeruginosa* clinical infections.

## Materials and methods

### Chemicals, media, bacterial strain and treatments

Geraniol (purity = 98%; relative density = 0.88 g/mL) was purchased from Shanghai Aladdin Bio-Chem Technology Co. Ltd. (Shanghai, China). The *P. aeruginosa* PAO1 strain was provided by the South China Sea Institute of Oceanology, Chinese Academy of Sciences, Guangzhou, China. *P. aeruginosa* PAO1 was cultured in Luria–Bertani (LB) broth (0.5% yeast extract, 1% tryptone, and 1% NaCl) with varying concentrations of geraniol (0, 0.275, 0.55, 1.1, and 2.2 mg/mL) at 180 rpm and 37°C.

### Transcriptomic analysis

Exponential growth phase *P. aeruginosa* PAO1 cells (1–2 × 10^8^ CFU/mL) of 1 mL were inoculated in 100 mL of fresh LB medium containing 0 (control groups) and 1.1 mg/mL (treatment groups) geraniol with three biological replicates, resulting in an initial cell concentration of 1–2 × 10^6^ CFU/mL. All six samples were incubated at 37°C and 180 rpm for 5 h. Then, the cell precipitates were collected, snap-frozen in liquid nitrogen, and stored at −80°C for RNA extraction.

The RNA extraction, library construction, and transcriptome sequencing and analysis were performed by Novogene Bioinformatics Technology Co. Ltd. (Beijing, China) as described in our previous work (Li et al., 2019). The raw data in fastq format were submitted to the National Center for Biotechnology Information (NCBI) Gene Expression Omnibus database (GSE216316). Differential expression analysis of the two groups (control and geraniol treatment) was performed using the DESeq2 R package. Genes with an adjusted *p*-value (padj) < 0.05 and |log2(foldchange)| > 1 were assigned as differentially expressed.

### qRT-PCR validation of key QS and virulence genes of the PAO1 strain

Differentially expressed genes (DEGs) involved in QS systems were selected for validation using qRT-PCR. The bacterial culture, geraniol treatment, and bacterial precipitate collection methods were the same as above, for transcriptome sequencing and analysis. *P. aeruginosa* PAO1 cells and geraniol concentrations were 1–2 × 10^6^ CFU/mL and 0 (control groups) or 1.1 mg/mL (treatment groups), respectively. Cells were incubated at 37°C and 180 rpm for 5 h. Total RNA extraction, RNA concentration and quality assessment, reverse transcription PCR (RT-PCR), and quantitative real-time PCR (qPCR) were performed as described in our previous work (Li et al., 2018). The primer information is provided in Supplementary Table S1.

### PAO1 growth and virulence factor determination

#### Growth inhibition assay

*Pseudomonas aeruginosa* PAO1 in the exponential growth phase at an initial concentration of 1–2 × 10^6^ CFU/mL were added to a 96-well microtiter plate containing geraniol (0, 2.2, 4.4, 8.8, 17.6, 35.2, 70.4, 140.8, and 281.6 mg/mL). The cultures were incubated at 37°C for 16–20 h to determine the minimum inhibitory concentration (MIC) of geraniol against *P. aeruginosa* PAO1.

*P. aeruginosa* PAO1 in the exponential growth phase at an initial concentration of 1–2 × 10^6^ CFU/mL were added to a 96-well microtiter plate containing geraniol (0, 0.28, 0.55, 1.1, and 2.2 mg/mL) in 200 μL of LB. The plate was incubated at 37°C and 180 rpm and the optical density (OD_600_) was recorded using a Spark® multimode microplate reader (Tecan, Swiss Confederation) at hourly intervals for up to 48 h.

#### PQS production assay

Exponential growth phase *P. aeruginosa* PAO1 cells (1 mL, 1–2 × 10^8^ CFU/mL) were inoculated in 100 mL LB medium with 0 and 1.1 mg/mL geraniol and incubated at 37°C and 180 rpm for 72 h. During the culture period, cell culture was collected at 24, 48, and 72 h, centrifuged at 12,000× *g* for 5 min, and the supernatant collected. The extraction and quantification of PQS was performed as described in a previous study (Li et al., 2020). A standard curve was formed with the concentration of the PQS standard sample and its peak area, and the PQS content in the experimental group was calculated according to its peak area.

#### Elastase activity assay

The exponential growth phase *P. aeruginosa* PAO1 cells (1 mL, 1–2 × 10^8^ CFU/mL) were inoculated in 100 mL LB medium with geraniol (0, 0.28, 0.55, 1.1, and 2.2 mg/mL) and incubated at 37°C and 180 rpm for 24 h. The 0 mg/mL geraniol group was set as control (+) and sterilized deionized water was used as control (−) instead of the PAO1 cell suspension. After incubation for 5 and 24 h, a 5 mL culture sample was prepared. The elastase activity assay was performed as described in a previous study (Li et al., 2018). The OD_495_ value was measured to determine elastase activity.

#### Pyocyanin assay

The exponential growth phase *P. aeruginosa* PAO1 cells (1 mL, 1–2 × 10^8^ CFU/mL) were inoculated in 100 mL LB medium with geraniol (0, 0.28, 0.55, 1.1, and 2.2 mg/mL) and incubated at 37°C and 180 rpm for 7 days. Sterilized deionized water was used as the negative control instead of the PAO1 cell suspension. A 5-mL culture was sampled each day and the pyocyanin assay was performed as described in a previous study (Li et al., 2018). The concentration of pyocyanin (μg/mL) was determined by multiplying the OD_520_ reading by 17.072 (Essar et al., 1990).

#### Biofilm assay

The effect of geraniol on biofilm formation was evaluated using 96-well microtiter plates as described in our previous work (Li et al., 2018). Two-hundred microliter samples of *P. aeruginosa* PAO1 cells during the exponential growth phase (initial concentration 1–2 × 10^6^ CFU/mL), geraniol (0, 0.28, 0.55, 1.1, and 2.2 mg/mL), and LB were added to a 96-well plate and incubated at 37°C for 24 h without agitation. Sterilized deionized water was used as the negative control instead of the PAO1 cell suspension. The OD_600_ and OD_590_ values were measured to obtain the concentration of cells in suspension and the biofilm yield, respectively.

### Statistical analysis

All of the experiments were performed in triplicate, and were repeated three times except for the transcriptomic sequencing. Statistical analysis was performed using IBM SPSS Statistics 25.0 software. The statistical data were presented as mean ± standard deviation (SD) and examined by a one-way analysis of variance followed by the Student–Newman–Keuls test. *p* < 0.05 and *p* < 0.01 indicates significant differences and extremely significant differences, respectively.

## Results

### Transcriptomic analysis

**Figure 2:**
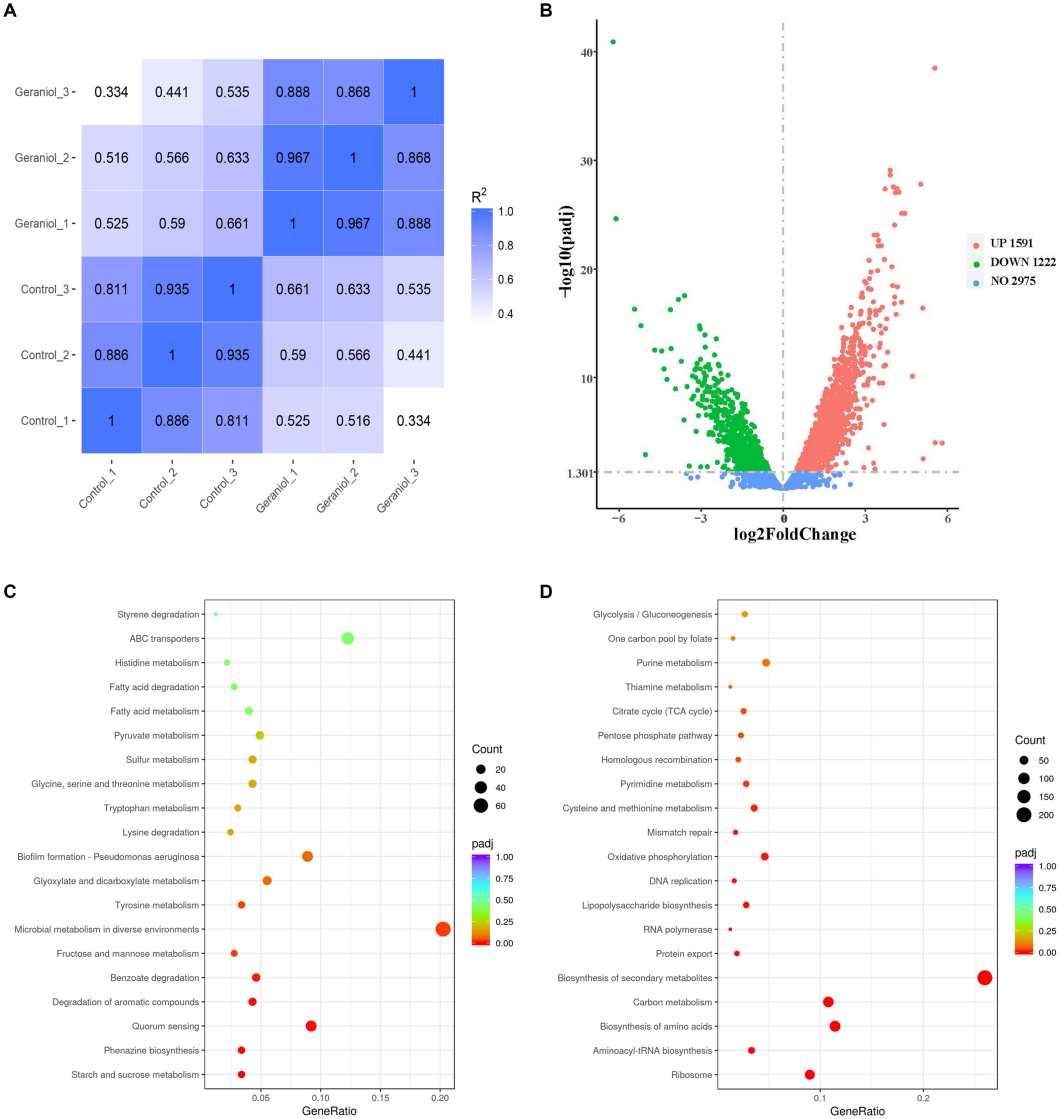
Maps of Pearson correlation coefficients, volcano plots, and Kyoto Encyclopedia of Genes and Genomes (KEGG) enrichment pathways based on RNA-seq data of three control and three treatment groups of *Pseudomonas aeruginosa* PAO1 with 1.1 mg/mL geraniol (0.00 < *q* < 0.05). **(A)** Pearson correlation coefficients map. **(B)** Volcano plots of differentially expressed genes. The red and green dots represent upregulated genes and downregulated genes with >2-fold change, respectively. **(C)** Bubble map of the top 20 KEGG enrichment pathways of downregulated genes with >2-fold change. **(D)** Bubble map of the top 20 KEGG enrichment pathways of upregulated genes with >2-fold change.

Transcriptomic sequencing was performed to examine the gene expression patterns of PAO1 exposed to 0 and 1.1 mg/mL geraniol. A map of the Pearson correlation coefficients is shown in Figure 2A. Pearson correlation coefficients showed that the correlation coefficient R2 of both the three control and three geraniol treatment groups was more than 0.8, and the R2 between control groups and geraniol treatment groups was less than 0.8. Therefore, transcriptomic data have intragroup biological repeatability and intergroup differences. A map of the volcano plots is shown in Figure 2B. The volcano plots detected a total of 5,788 genes based on RNA-seq of *P. aeruginosa* PAO1 cells which were treated and untreated with geraniol, of which 852 genes were significantly downregulated, 1,169 genes were significantly upregulated with fold change >2 and padj <0.05, and 3,767 genes did not exhibit altered expression levels.

The bubble maps of the top 20 Kyto Encyclopedia of Genes and Genomes (KEGG) enrichment pathways of downregulated and upregulated genes with >2-fold changes are shown in Figures 2C,D, respectively. Figure 2C shows that starch and sucrose metabolism, phenazine biosynthesis, QS, degradation of aromatic compounds, benzoate degradation, fructose and mannose metabolism, microbial metabolism in diverse environments, tyrosine metabolism, glyoxylate and dicarboxylate metabolism, and biofilm formation were the top ten pathways enriched with significantly downregulated genes after geraniol treatment. In these downregulated pathways, phenazine biosynthesis, QS, and biofilm formation pathways were enriched with key genes involved in QS and virulence genes regulated by QS. The detailed QS, phenazine biosynthesis, and biofilm formation pathways are shown in Supplementary Figures S1A–C, respectively, and the transcriptional changes of key genes involved in the three pathways are shown in Table 1.

**Table 1:** Differentially expressed key genes involved in *Pseudomonas aeruginosa* PAO1 quorum sensing systems and the related virulence genes based on RNA-seq.

Gene id	Gene name	Log_2_^FoldChange^	padj	Pathway name
PA1432	*lasI*	1.289060024	0.005788915	Quorum sensing
PA1430	*lasR*	−0.521916465	0.296199632	Quorum sensing
PA3476	*rhlI*	1.816412165	2.45474E-05	Quorum sensing
PA3477	*rhlR*	−0.798945862	0.161883353	Quorum sensing
PA0996	*pqsA*	−1.632334557	5.66601E-05	Quorum sensing
PA0997	*pqsB*	−1.857914814	1.84251E-08	Quorum sensing
PA0998	*pqsC*	−1.867542731	1.35379E-09	Quorum sensing
PA0999	*pqsD*	−1.474298994	5.46105E-06	Quorum sensing
PA1000	*pqsE*	−1.098343318	0.000159382	Quorum sensing
PA1001	*phnA*	−1.289316266	3.80E-05	Quorum sensing
PA1002	*phnB*	−0.961817481	0.001568692	Quorum sensing
PA1003	*pqsR* (*mvfR*)	0.12379533	0.785649354	Quorum sensing
PA2587	*pqsH*	0.054480343	0.906288652	Quorum sensing
PA3479	*rhlA*	−2.161753902	7.61774E-05	Quorum sensing
PA3478	*rhlB*	−1.879839834	4.79516E-05	Quorum sensing
PA1130	*rhlC*	−1.42583676	9.6008E-06	Quorum sensing
PA1871	*lasA*	−3.16297037	1.3179E-11	Quorum sensing
PA3724	*lasB*	−1.94856245	8.18372E-06	Quorum sensing
PA2570	*lecA*	−5.189666242	1.70617E-15	Quorum sensing
PA3361	*lecB*	−6.205171887	1.30495E-41	Quorum sensing
PA3064	*pelA*	−0.928811017	0.04059119	Biofilm formation
PA3063	*pelB*	−1.063973108	0.040195695	Biofilm formation
PA3058	*pelG*	−1.151061762	0.008035269	Biofilm formation
PA1900	*phzB2*	−2.467137917	1.84251E-08	Phenazine biosynthesis
PA4209	*phzM*	−1.757052575	2.03581E-06	Phenazine biosynthesis
PA4217	*phzS*	−1.743599304	3.05E-09	Phenazine biosynthesis

The QS pathway of PAO1 was significantly downregulated after geraniol treatment according to the above results. Table 1 and Supplementary Figure S1A show the changes in the transcriptional levels of key genes involved in the PAO1 QS systems after geraniol treatment. In the *las* system, the *lasI* gene, encoding the 3OC_12_-HSL signal synthase LasI, was significantly upregulated, whereas expression of the *lasR* gene, encoding the signal receptor LasR, was downregulated (<2-fold change). Similarly, in the *rhl* system, the *rhlI* gene, which encodes the C_4_-HSL signal synthase RhlI, was significantly upregulated, and the expression of *rhlR*, which encodes the signal receptor RhlR, was downregulated (< 2-fold change). In the *pqs* system, *pqsABCDE* genes, which are involved in PQS biosynthesis, were significantly downregulated (> 2-fold change), *pqsH* expression was unchanged, and the expression of *pqsR*, encoding the PQS signal receptor protein, was also unchanged. Table 1 and Supplementary Figure S1A also show changes in the expression levels of some virulence genes regulated by the QS systems. After geraniol treatment, *lasA* (encoding protease LasA), *lasB* (encoding elastase LasB), *lecA* (encoding lectin LecA), *lecB* (encoding lectin LecB), and *rhlABC* (encoding rhamnolipids) expression levels were all downregulated.

Changes in the transcriptional levels of key genes involved in phenazine biosynthesis in the PAO1 strain after geraniol treatment are shown in Table 1 and Supplementary Figure S1B. Most key genes involved in phenazine biosynthesis (including pyocyanin) were downregulated, such as *phzAB*, *phzM*, and *phzS*.

Changes in the transcriptional levels of key genes involved in biofilm formation of the PAO1 strain after geraniol treatment are shown in Table 1 and Supplementary Figure S1C. Some genes involved in biofilm formation were downregulated, such as Pel polysaccharide encoding genes, *pelAB* and *pelG*; rhamnolipid encoding genes *rhlABC*, and lectin encoding genes *lecA* and *lecB*.

The top ten KEGG pathways significantly enriched with upregulated genes after geraniol treatment included ribosome, aminoacyl-tRNA biosynthesis, biosynthesis of amino acids, carbon metabolism, biosynthesis of secondary metabolites, protein export, RNA polymerase, lipopolysaccharide biosynthesis, DNA replication, and oxidative phosphorylation (Figure 2D). These upregulated pathways were not related to QS systems or their related virulence genes.

### qRT-PCR to validate the results of the transcriptomic analysis

**Figure 3:**
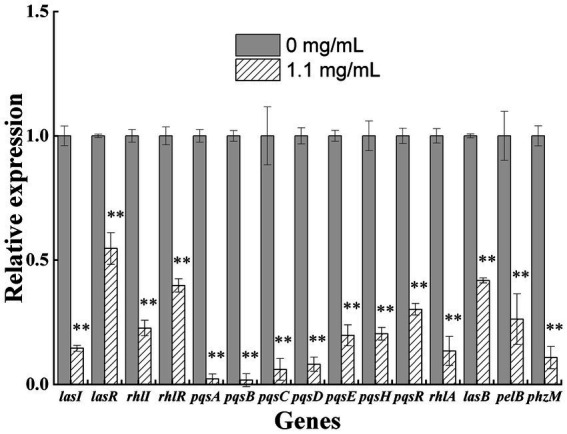
Quantitative reverse transcription polymerase chain reaction validation map of some differentially expressed key genes in the quorum sensing systems and virulence genes of *Pseudomonas aeruginosa* PAO1 exposed to 1.1 mg/ml geraniol. Independent Student’s *t*-tests were performed to compare the control and geraniol treatment groups. *p < 0.05; **p < 0.01.

PAO1 cells were treated with 1.1 mg/mL geraniol for 5 h, and the expression levels of key genes in the QS systems and related virulence genes were validated using qRT-PCR (Figure 3). Eleven key QS genes (*lasI*, *lasR*, *rhlI*, *rhlR*, *pqsABCDEH*, and *pqsR*) and four virulence genes (*rhlA*, *lasB*, *pelB*, and *phzM*) were selected for validation. In the *las* system, *lasI* (>2-fold change) and *lasR* (<2-fold change) were significantly downregulated; in the *rhl* system, *rhlI* and *rhlR* were significantly downregulated (>2-fold change); in the *pqs* system, *pqsABCDEH* and *pqsR* were significantly downregulated (>2-fold change). Therefore, the gene expression trends of key genes in the QS systems were consistent with the transcriptomic results. Similarly, the expression levels of the virulence genes were consistent with those of the transcriptomic results, with *rhlA*, *lasB*, *pelB*, and *phzM* being significantly downregulated (>2-fold change).

### PAO1 growth and virulence factors

#### Growth curve

**Figure 4:**
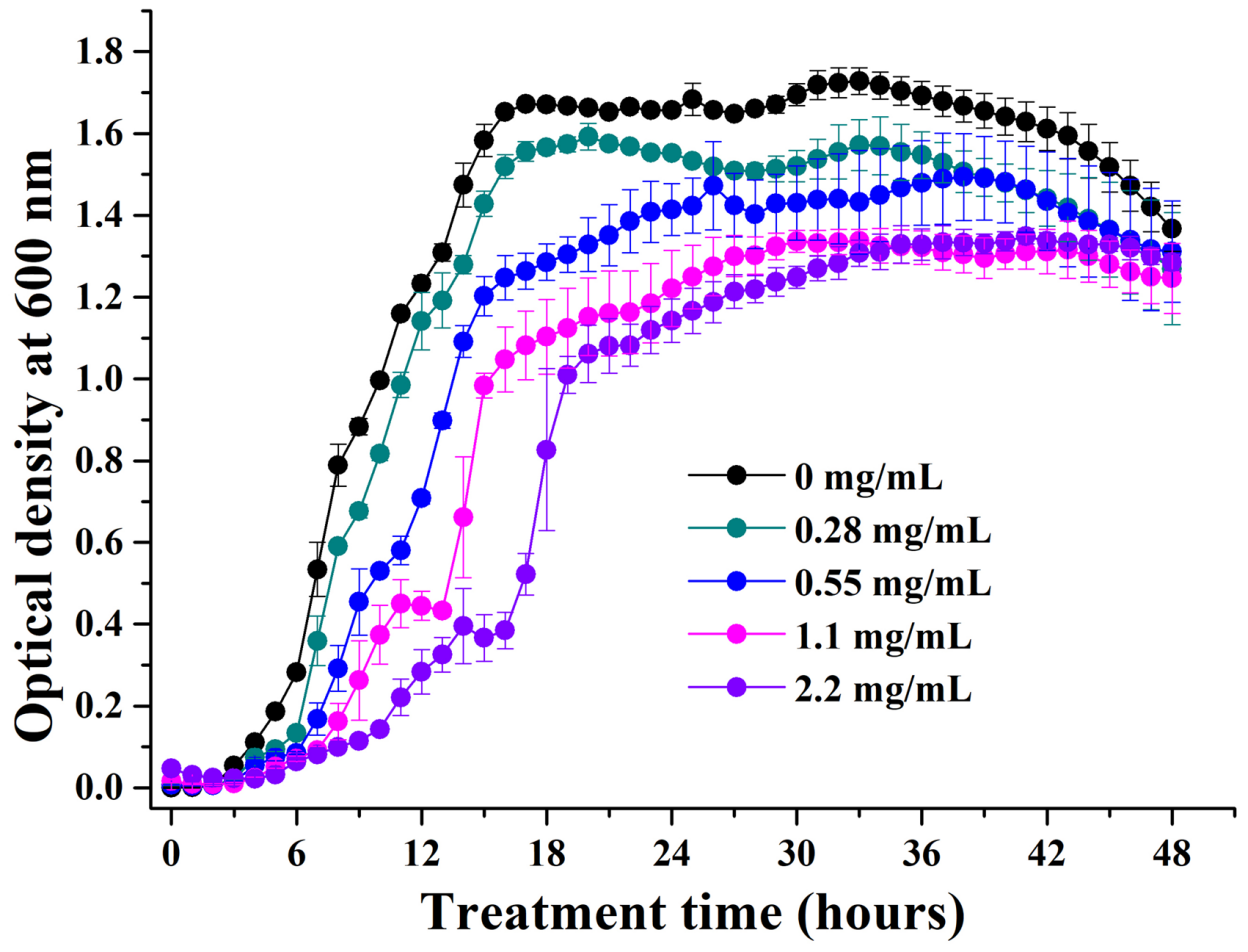
Growth curves of *Pseudomonas aeruginosa* PAO1 treated with different concentrations of geraniol.

The growth inhibition experiments showed that the MIC of geraniol against *P. aeruginosa* PAO1 was 70.4 mg/mL. The growth curves of PAO1 cells treated with different geraniol concentrations were drawn based on the absorbance values at 600 nm for 48 h. PAO1 growth of the control group showed a typical growth curve, including lag, exponential, and stationary phases (Figure 4). The growth curve did not show a decline phase because the OD_600_ reflected the total number of living and dead cells. PAO1 cell growth in different geraniol treatment groups showed a prolonged lag phase, delayed growth periods, and reduced growth to different degrees compared to the control group. However, the main PAO1 population cells were not inhibited by geraniol. Therefore, geraniol slightly affected *P. aeruginosa* growth.

#### PQS production

**Figure 5:**
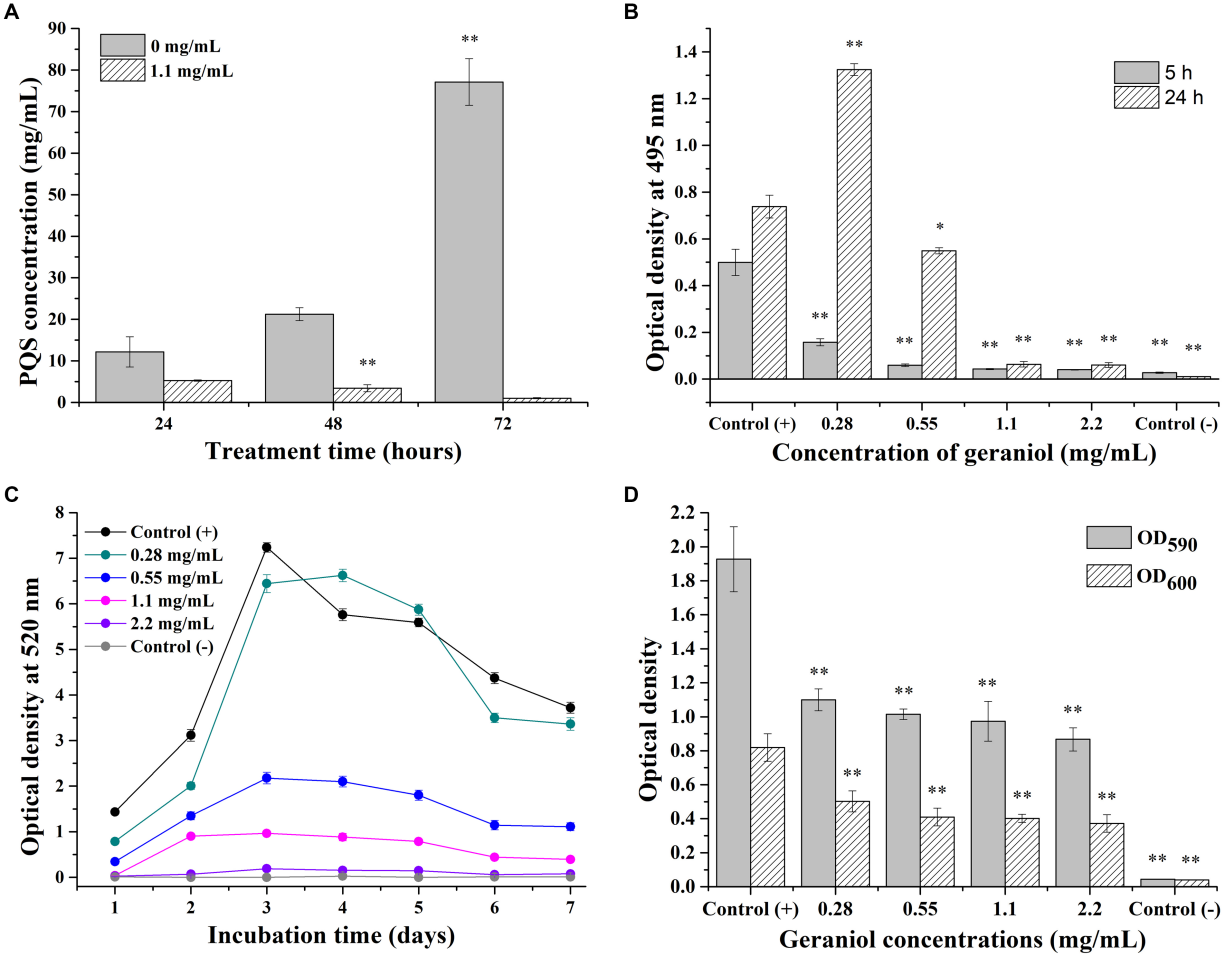
Maps presenting the effect of geraniol on *Pseudomonas aeruginosa* PAO1 virulence factors. **(A)** Histogram of *Pseudomonas* quinolone signal production. **(B)** Histogram of elastase LasB production. **(C)** Curves of pyocyanin production. **(D)** Histogram of biofilm and planktonic cell yield. OD_600_ and OD_590_ mean relative planktonic cell concentration and biofilm yield, respectively. Independent Student’s *t*-tests were performed to compare the control and geraniol treatment groups. *p < 0.05; **p < 0.01.

The changes in PQS production after PAO1 cells were treated with 1.1 mg/mL geraniol are shown in Figure 5A. The PQS production increased with increasing incubation time in the control group, with the highest concentration at 72 h of incubation and the lowest concentration at 24 h of incubation. The PQS production in the geraniol treatment group did not show a trend of increasing production with an increase in incubation time as compared to the control group. The PQS concentrations in the geraniol treatment group were significantly decreased compared to those in the control group after incubation for 48 and 72 h. Therefore, geraniol significantly inhibited the PQS production ability of *P. aeruginosa* PAO1.

#### Elastase LasB activity

PAO1 cells were treated with different concentrations of geraniol and the elastase LasB activity in the culture supernatants were determined at 5 and 24 h (Figure 5B). In the control group, elastase LasB production increased with increasing incubation time, with the elastase production at 24 h of incubation being higher than that at 5 h. The elastase production in the geraniol treatment groups (0.55–2.2 mg/mL) were significantly decreased compared with that of the control group and did not show an increase with the increased incubation time. However, in the 0.28 mg/mL geraniol treatment group, elastase production increased with increasing incubation time, and elastase production after 24 h of incubation was significantly higher than that in the control group. This indicated that a low concentration geraniol (0.28 mg/ml) stimulates elastase production in the PAO1 strain. Therefore, geraniol significantly inhibited elastase production by *P. aeruginosa* PAO1 in a concentration-dependent manner, except at low concentrations (0.28 mg/ml).

#### Pyocyanin yield

The production of pyocyanin by PAO1 cells after continuous treatment with different geraniol concentrations for 7 days is presented in Figure 5C. In the control (+) group, pyocyanin production increased continuously during the first 3 days of incubation and reached a maximum on the third day of incubation. In the next 4 days of incubation, pyocyanin production in the control group decreased continuously. In the control (−) group, pyocyanin production in cell-free cultures was nearly zero after incubation for one to 7 days. The pyocyanin production trends in different geraniol treatment groups (0.28–2.2 mg/mL) were consistent with that of the control. However, pyocyanin yields decreased with increasing geraniol concentration. Especially in the 2.2 mg/mL geraniol treatment group, pyocyanin production was similar to that in the control (−) group, nearly zero after 7 days of incubation. Therefore, geraniol attenuated pyocyanin production by *P. aeruginosa* PAO1 in a concentration-dependent manner.

#### Biofilm formation

PAO1 cells were treated with different concentrations of geraniol, seeded in a 96-well plate, and incubated for 24 h. The relative bacterial-suspension concentration (OD_600_) and biofilm yield (OD_590_) were determined (Figure 5D). The relative PAO1 cell-suspension concentration and biofilm yield were normal in the control (+) group, while the OD_600_ and OD_590_ were nearly zero in the control (−) group. The bacterial-suspension concentration and biofilm yield both decreased in all geraniol treatment groups (0.28–2.2 mg/mL) compared with that of the control (+) group. Therefore, geraniol inhibited planktonic *P. aeruginosa* PAO1 cells growth and biofilm formation.

## Discussion

Geraniol, a chemical component of citronella, ginger, lemon, rose, nutmeg, lavender, and orange essential oils, is a colorless to pale-yellow liquid with a rose-like scent (Silva et al., 2022). The growth curves of *P. aeruginosa* PAO1 cells treated with geraniol showed that geraniol slightly affected cell growth, prolongs the lag phase, and delays the growth period, but with the main population of cells not being inhibited. Another study found that *Palmarosa* essential oil, which is composed of 81.25% geraniol, had a slight antibacterial activity against *P. aeruginosa* PAO1 growth and inhibited certain virulence factors regulated by the QS systems (Önem, 2022).

Transcriptomic analysis detected a total of 5,788 genes, of which 852 and 1,169 genes were significantly downregulated and upregulated, respectively. The KEGG pathways of QS, biofilm formation, and phenazine biosynthesis were enriched with the downregulated key genes involved in *P. aeruginosa* QS systems and virulence factor encoding genes regulated by the QS systems. These transcriptomic and qRT-PCR results showed that geraniol inhibited *lasI* expression, which encodes 3OC_12_-HSL signal synthetase in the las system, *rhlI* expression, which encodes C_4_-HSL signal synthetase in the *rhl* system, and *pqsABCDEH* expression, which are involved in PQS biosynthesis in the *pqs* system, and inhibited the expression of *lasR*, *rhlR*, and *pqsR* encoding three corresponding signal receptor proteins, respectively.

Moreover, some virulence genes, including *rhlABC* involved in rhamnolipid biosynthesis; *lasA* and *lasB* encoding exoprotease LasA and elastase LasB, respectively; *lecA* encoding lectin LecA and *lecB* encoding lectin LecB; *pelAB* and *pelG* involved in biofilm formation; and *phzAB*, *phzM*, and *phzS* involved in pyocyanin biosynthesis were all downregulated by geraniol as shown in the transcriptomic results. The qRT-PCR results showed similar down transcriptional trends to those of the transcriptomic analysis. Furthermore, the physiological and biochemical results showed similar downregulation trends upon geraniol exposure, with a decrease in PQS signals, elastase, pyocyanin, and biofilm yields.

The transcriptomic, qRT-PCR, and physiological-biochemical results showed that geraniol inhibited 3OC_12_-HSL, C_4_-HSL, and PQS signal biosynthesis and the corresponding receptor proteins, resulting in the attenuation of some virulence factors regulated by the three QS systems, such as exoprotease LasA, elastase, lectin, rhamnolipids, pyocyanin, and biofilm formation. These results indicate that the inhibition of the three QS systems could attenuate the virulence factors indirectly regulated by the QS systems. Some previous studies on the inhibition of *P. aeruginosa* PAO1 QS systems by diallyl sulfide (Li W. R. et al., 2021), diallyl disulfide (Li et al., 2019), diallyl trisulfide (Li et al., 2022), and farnesol had similar findings (Li et al., 2020). Diallyl sulfide, diallyl disulfide, diallyl trisulfide, and geraniol inhibited the three QS systems of *las*, *rhl*, and *pqs*. Farnesol inhibited key genes involved in PQS biosynthesis of the *pqs* system but did not affect the expression levels of *pqsR* of the *pqs* system, *lasI*/*lasR* of the *las* system, and *rhlI*/*rhlR* of the *rhl* system (Li et al., 2020). Nonetheless, geraniol, diallyl sulfide, diallyl disulfide, diallyl trisulfide, and farnesol inhibited the transcription of the relevant virulence genes regulated by the three QS systems. A previous study also reported that a null mutation in the *pqs* system induced a decrease in the production of biofilms, pyocyanin, elastase, lectin, and rhamnolipids (Cao et al., 2001; Diggle et al., 2003). The *pqs* signaling system is important in the biosynthesis regulation of the virulence factors mentioned above.

In conclusion, the MIC of geraniol against *P. aeruginosa* PAO1 was 70.4 mg/ml. Geraniol of 1.1 mg/ml slightly affected PAO1 growth, prolonged the lag phase, and delayed the growth periods. However, geraniol inhibited the three *P. aeruginosa* QS systems, *las*, *rhl*, and *pqs* by suppressing the expression level of their key genes, including *lasI*, *rhlI*, *pqsABCDEH*, *lasR*, *rhlR*, and *pqsR*. Geraniol also suppressed certain virulence genes regulated by the three QS systems, including *rhlABC*, *lasAB*, *lecAB*, *phzABMS*, and *pelABG*, resulting in the attenuation of related virulence factors of rhamnolipids, exoproteases, lectins, pyocyanin, and biofilm. Therefore, geraniol suppresses *P. aeruginosa* PAO1 virulence by inhibiting its three QS systems of *las*, *rhl*, and *pqs*.

## Data availability statement

The datasets presented in this study can be found in online repositories. The names of the repository/repositories and accession number(s) can be found in the article/Supplementary material.

## Author contributions

W-RL and T-HZ: conceptualization, formal analysis, investigation, and writing–review and editing. W-RL, T-HZ, and Z-QZ: methodology and validation. X-BX and Q-SS: supervision. W-RL: funding acquisition and project administration. All authors have read and agreed to the published version of the manuscript.

## Funding

This research was funded by Guangdong Basic and Applied Basic Research Foundation (2021A1515011080 and 2020A1515010850), and GDAS’ Project of Science and Technology Development (2018GDASCX-0102).

## Conflict of interest

The authors declare that the research was conducted in the absence of any commercial or financial relationships that could be construed as a potential conflict of interest.

## Publisher’s note

All claims expressed in this article are solely those of the authors and do not necessarily represent those of their affiliated organizations, or those of the publisher, the editors and the reviewers. Any product that may be evaluated in this article, or claim that may be made by its manufacturer, is not guaranteed or endorsed by the publisher.
