# Supplementary material for: Geraniol attenuates virulence factors by inhibiting quorum sensing of Pseudomonas aeruginosa
Source: Front Microbiol. 2023 Apr 27;14:1190619. doi: 10.3389/fmicb.2023.1190619 (PMC10172488; doi:10.3389/fmicb.2023.1190619)
Supplement: Supplementary file 1 [file Data_Sheet_1.docx]

**Geraniol attenuates virulence factors via inhibiting quorum sensing of *Pseudomonas aeruginosa***

Wen-Ru Li, Tao-Hua Zeng, Zhi-Qing Zhang, Qing-Shan Shi, Xiao-Bao Xie^*^

Key Laboratory of Agricultural Microbiomics and Precision Application (MARA), Guangdong Provincial Key Laboratory of Microbial Culture Collection and Application, Key Laboratory of Agricultural Microbiome (MARA), State Key Laboratory of Applied Microbiology Southern China, Institute of Microbiology, Guangdong Academy of Sciences, Guangzhou 510070, China

^*^Corresponding author

Tel: +86-20-37656986, Fax: +86-20-37656986

E-mail: [xiexb@gdim.cn](mailto:xiexb@gdim.cn)

**Table S1 The primer information used for real time PCR.**

| **Gene name** | **Locus tag** | **Primer sequence (5′–3′)** | **primer design** |
| --- | --- | --- | --- |
| 16S rRNA | PA5369.5 | GCGCAACCCTTGTCCTTAGTT(F) | (Li et al., 2018)  (Li et al., 2018)  (Li et al., 2018)  (Li et al., 2018)  (Li et al., 2018)  Designed using Primer Premier 6.0  Designed using Primer Premier 6.0  Designed using Primer Premier 6.0  Designed using Primer Premier 6.0  Designed using Primer Premier 6.0  Designed using Primer Premier 6.0  Designed using Primer Premier 6.0  (Li et al., 2018)  Designed using Primer Premier 6.0  Designed using Primer Premier 6.0  (Li et al., 2020) |
|  |  | TGTCACCGGCAGTCTCCTTAG(R) |  |
| *lasI* | PA1432 | TGCGTGCTCAAGTGTTCAAGG(F) |  |
|  |  | CGGCTGAGTTCCCAGATGTGC (R) |  |
| *lasR* | PA1430 | GACCAGTTGGGAGATATCGGTTA(F) |  |
|  |  | TCCGCCGAATATTTCCCATA(R) |  |
| *rhlI* | PA3476 | AAACCCGCTACATCGTCGC (F) |  |
|  |  | TCTCGCCCTTGACCTTCTGC(R) |  |
| *rhlR* | PA3477 | ATCGCCATCATCCTGAGCATT(F) |  |
|  |  | TCGGAGGACATACCAGCACAC(R) |  |
| *pqsA* | PA0996 | GTTGCCTGGCTATGAGTG (F) |  |
|  |  | ACCTTGAACAGATCGTCTTC (R) |  |
| *pqsB* | PA0997 | CCGATATTCGCCAGGTAG (F) |  |
|  |  | ATCAGCAGCAGTTCATCC(R) |  |
| *pqsC* | PA0998 | GGATTCGCAGATGGAGTG(F) |  |
|  |  | GAGCAGGTTGGAGATGTAC(R) |  |
| *pqsD* | PA0999 | CCTTCCTCGACGAGAATG(F) |  |
|  |  | GTTCGGTTGATGGCAGAT(R) |  |
| *pqsE* | PA1000 | GACGACATGGAGGCTTAC(F) |  |
|  |  | CGCAGACACTCGGTATAG(R) |  |
| *pqsH* | PA2587 | GTAGTGCTGATCGGTGAC(F) |  |
|  |  | GATTGCTTCCTGATGAACTC(R) |  |
| *pqsR* | PA1003 | GTTCTGCGATACGGTGAG(F) |  |
|  |  | CGATGGTGATGGCGATAT(R) |  |
| *lasB* | PA3724 | AAGGCCTTGCGGGTATCC(F) |  |
|  |  | CGTGTACAACCGTGCGTTCT(R) |  |
| *rhlA* | PA3479 | CAGCAACCATCAGCACAT (F) |  |
|  |  | TCCAGGCAAGCCAAGTAG (R) |  |
| *pelB* | PA3063 | CGCTGAGTGTCAAGGAAC (F) |  |
|  |  | CTGCCAACTGCCGATAAG (R) |  |
| *phzM* | PA4209 | GAATGGAAGTCCCGTTGC(F) |  |
|  |  | GCCCTCGACATCCCTCA(R) |  |


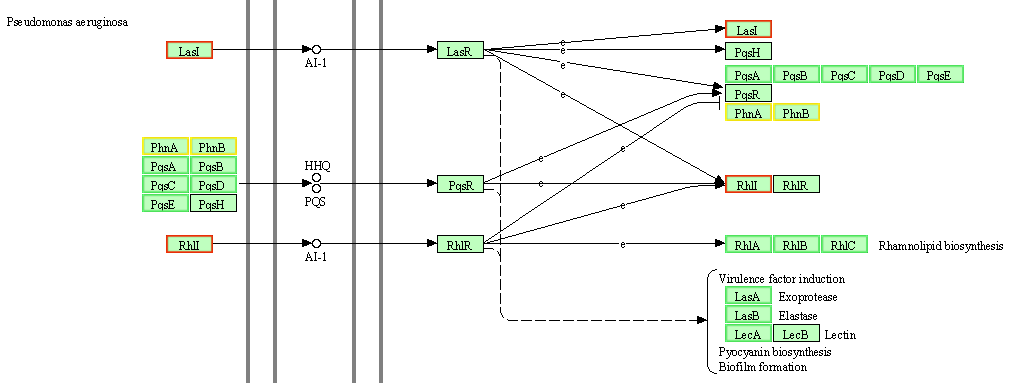


**A**

**B**


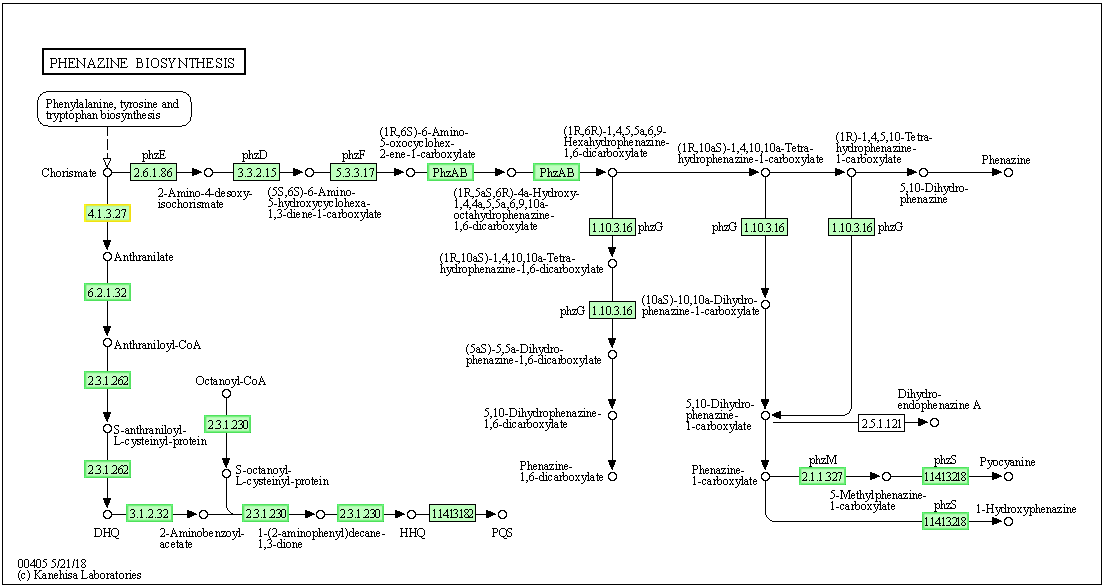


**C**


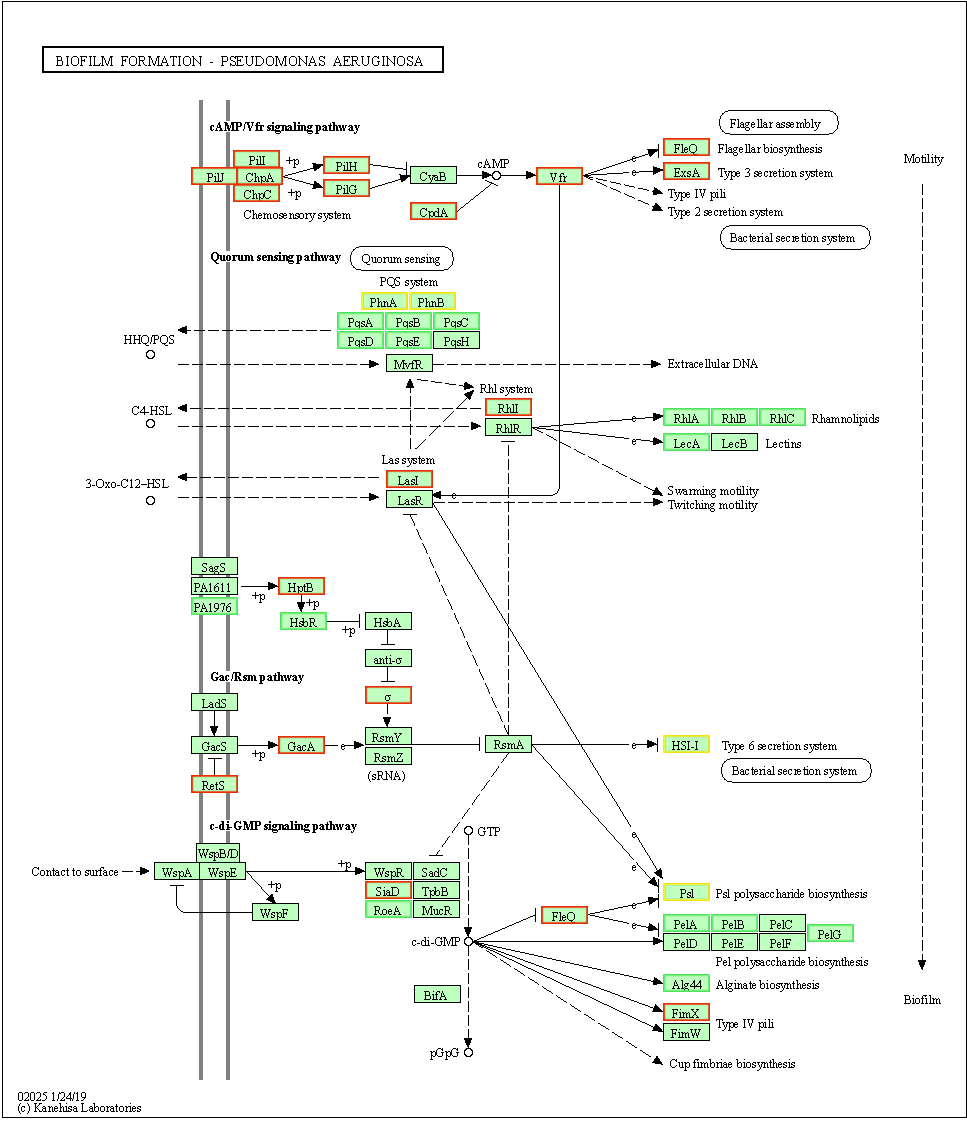


**Figure S1** The significantly enriched KEGG pathways based on RNA-seq analysis of Pseudomonas aeruginosa PAO1 (control group vs geraniol-treated group). RNA in blue boxes, red boxes, and black boxes indicated that their transcriptional levels were downregulated, upregulated, and no changed, respectively. A, quorum sensing; B, phenazine biosynthesis; C, biofilm formation.
